# Structural evolution of in situ polymerized poly(L-lactic acid) nanocomposite for smart textile application

**DOI:** 10.1038/s41598-022-17437-z

**Published:** 2022-08-30

**Authors:** Doli Hazarika, Amit Kumar, Vimal Katiyar

**Affiliations:** Department of Chemical Engineering, Indian Institute of Technology Guwahati, Assam, 781039 India

**Keywords:** Engineering, Nanoscience and technology

## Abstract

This present study demonstrated the preparation of a highly crystalline anatase (ana) form of titanium oxide (TiO_2_) doped silk nanocrystal (SNC) nanohybrid (ana-TCS) of diameter (7.5 ± 1.4 nm) by the sol–gel method using titanium (IV) butoxide as the hydrolysis material. This prepared nanohybrid with surface hydroxyl groups acted as a co-initiator for the synthesis of poly(L-lactic acid) (PLLA)-g-ana-TSC nanocomposite with grafted PLLA chains via the in situ polymerization technique, using tin-octoate as a catalyst. The fabricated nanocomposite had a high number average molecular weight of 83 kDa with good processibility. This prepared nanocomposite was hydrophobic in nature, with a contact angle of 105°, which was further enhanced to 122 ± 1° when processed via electrospinning technique into a non-woven fabric. The prepared nanocomposite could degrade up to 43% methylene blue dye in 15 days. This nanocomposite showed no significant molecular weight reduction after 1 h of aqeous treatment, which could be attributed to its hydrophobic nature, inhibiting its degradation. However, 50% degradation was observed for the nanocomoposite whereas, PLLA demonstrated 25% degradation in 15 days, after its end-of-life. Thus, this study revealed that the in situ synthesized PLA-ana-TCS nanocomposite could be targeted for use as a hydrophobic, self-cleaning, dye-degradable fabric.

## Introduction

Rapid urbanization, industrialization, and fast population growth have resulted in enormous consumption of plastics for packaging, construction, and different household activities. These plastics are fossil fuel derived products and cannot be disposed easily after their end-of-life, as they are non-biodegradable, giving rise to serious environmental troubles by contaminating land, water as well as air. Biodegradable plastics are being looked upon as an alternative to these synthetic plastics due to their environmentally benign properties attained because of the presence of hydrolysable ester bonds that gets decomposed by microbial activity, thereby evading the use of thermal treatment techniques like incineration, which cause a lot of pollution^1^.

Poly(lactic acid) (PLA), a linear aliphatic thermoplastic polyester, derived from 100% renewable resources, has been hailed by many researchers as one of the most promising bioplastic, owing to its eco-friendly nature, biocompatibility, biodegradability^2^, and processability easiness^3^. PLA is synthesized on a large scale either by direct condensation of lactic acid or by ring-opening polymerization (ROP) of cyclic lactide, with the later approach being more favorable due its solvent free characteristic, resulting in no harmful byproduct formation^4^. PLA can be processed by film casting, extrusion, blow molding, or fiber spinning due to its greater thermal processability in comparison to other biomaterials such as poly(ethylene glycol), poly(hydroxyalkanoates), and poly(ɛ-caprolactone), leading to its widespread application in industries like textiles, biomedical and food packaging^5^. However, a few limitations of PLA such as low glass transition temperature, low melt strength, slow crystallization rate, low toughness, high brittleness, and low heat deflection temperature has made its use limited in broad scale engineering purposes^6^. Recent researches focus on using biofillers derived from plants and animals into the PLA matrix to improve its properties^7^. A recent study showed the effect of silk nanomaterial hydrolyzed from muga silk cocoons as a biofiller for the improvement of crystallinity and thermal stability of PLA^8^. These silk nanofillers originated from silk fibroin (SF) having repetitive and well-ordered β-sheets in their protein amorphous structure, displaying highly crystalline and hydrophobic properties. These properties make them useful as a stiffening material in textile industries due to the presence of strong hydrogen bonding. Another study showed the application of magnetized silk nano-discs prepared by the co-precipitation technique and melt blended with PLA for tissue engineering purposes^8,9^. Various studies have reported that doping of inorganic materials into organic nanomaterials can tune various properties of a polymer when incorporated into its matrix. Titanium dioxide (TiO_2_), a metal oxide, also known as titania, has excellent chemical, structure and thermal stability, and is also biocompatible and non-toxic in nature, having high catalytic activity and low cost^10^. Titania also possesses a unique self-cleaning property and can also function as an UV blocking agent^11^. Anatase (ana), one of the three crystallographic polymorph of titania, is highly photoactive in nature and had been used for the degradation of organic pollutants^12^. Different techniques like chemical precipitation, microemulsification, hydrothermal crystallization, solvo-thermal method, biological synthesis and sol–gel method have been utilized for the bottom down nanoscale synthesis of TiO_2_ to obtain high specific surface area. Out of the various techniques documented, sol–gel method has been established as one of the most successful strategies for obtaining uniformly dispersed nano-sized metallic oxide materials^13^. Previous studies had reported the synthesis of CNC (cellulose nanocrystal)-TiO_2_ colloidal nanohybrids as a core–shell structure to form a neutral surface^14^. Another study had discussed about novel regenerated SF/TiO_2_ nanocomposites synthesized by the sol–gel technique for improvement in their mechanical and thermal properties^15^.

Commercialization of the application of PLA in textile commodities have been limited as PLA is prone to UV damage when exposed to sunlight for a long time, thereby restricting its usefulness as an environment-friendly material. The current study depicts the use of a biofiller supported with a metal oxide in the matrix of the biodegradable polymer, poly(L-lactic acid) (PLLA) to improve the crystallinity, thermal stability, and hydrophobicity of the prepared fabric. A facile sol–gel method was applied for the synthesis of polycrystalline ana-TCS, wherein SNC was used as the template. This anatase TiO_2_ doped SNC nanohybrid acted as a nucleating agent and was incorporated into the PLLA matrix, resulting in the fabrication of a nanocomposite via a green synthesis route, i.e., in situ polymerization technique, which is essentially a solvent-free method. This is the first study describing the in situ fabrication of a biodegradable and compostable PLLA/ana-TCS nanocomposite, spun into a nanofabric, behaving as a smart textile.

## Results and discussion

### Molecular weight analysis

Molecular weight analysis reveals the chain distribution, which strongly depends on the synthesis parameters. GPC chromatogram of the fabricated nanocomposite has been shown in Supplementary Figure S2. The weight average molecular weight, *M*_w_ and number average molecular weight, *M*_n_ of the prepared nanocomposite were found to be 83 kDa and 150 kDa, respectively. A shoulder peak was seen near a high molecular weight position, signifying higher molecular weight grafted chains due to short and long branch formations^16^. The peak at elution time around 19.2 min appeared due to the mobile phase, chloroform and other moisture traces, if any.

### Structural analysis

**Figure 1:**
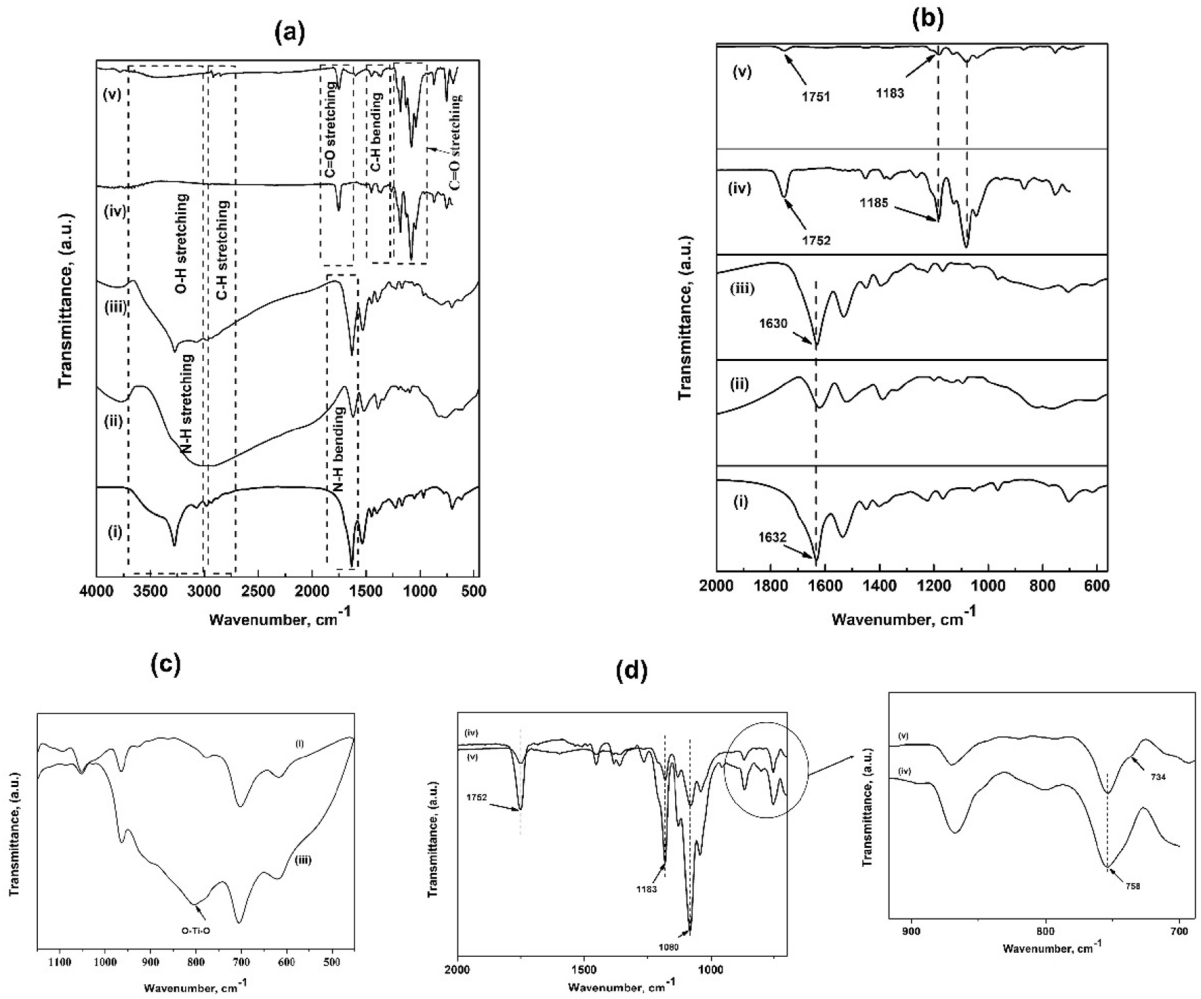
(**a**) FTIR spectra for (i) SNC; (ii) TCS; (iii) ana-TCS; (iv) PLLA (synthesized in lab for reference); (v) PLLA/ana-TCS, (**b**) Magnified FTIR spectra of the samples within the range of 2000–620 cm^−1^, (**c**) Formation of O–Ti–O linkage in ana-TCS, (**d**) FTIR spectra of PLLA/ana-TCS within the range of 2000–700 cm^-1^, showing the presence of O–Ti–O bond.

FTIR spectrum depicting the interfacial interaction of the lab prepared ana-TCS, and PLLA/ana-TCS nanocomposite has been shown in Fig. 1. The amorphous dried TCS powder gave broad peaks at 3340 cm^−1^ and 3017 cm^−1^, which were assigned to stretching O–H groups adsorbed on the surface of TiO_2_. After calcination, a band at 3274 cm^−1^ was observed due to the presence of unbonded N–H group of SNC, which was absent in as-prepared TCS due to the removal of adsorbed water molecules. The intense peaks found at 1695 cm^−1^, 1634 cm^−1^, and 705 cm^−1^ represented amide I due to their better arranged β structure in SNC after the removal of the amorphous part. Peak at 1534 cm^−1^ was for amide II (C–N) and peak at 1229 cm^-1^ was for amide III (C–N–H)^8,17^. For nanohybrid, the peak for NH– shifted to 1530 cm^−1^ and was found to be highly intensified, suggesting bond formation between NH and OH– of TiO_2_. Another slight shifting of the peak from 1632 to 1629 cm^–1^ indicated bond formation between Ti–OH of titania and –C=O from the peptide bonds of SNC. It had been reported that the signals within the range of 1000–400 cm^−1^ occurred due to the Ti–O, O–Ti–O and Ti–OH bending vibration bonds, however observation of new peaks around 806 cm^−1^ was assigned to O–Ti–O bond ^18^. In case of PLLA/ana-TCS, a band around 2916 cm^−1^ was assigned to aliphatic C–H vibrations. A decrease in intensity of C=O peak and appearance of a little hump due to –OH group of ana-TCS led to observation of bands at 1754 cm^−1^ and 1613 cm^−1^, respectively. For neat PLLA, ester bond (C=O) was observed at 1752 cm^−1^, asymmetric and symmetric C–O vibration bands were seen at 1182 and 1083 cm^−1^, respectively, whereas the symmetric and asymmetric vibrations of C–H were observed at 1452 and 1361 cm^−1^, respectively. The functional groups to confirm the grafting of ana-TCS with PLLA chains were established by shifting of PLLA chain groups assigned to C=O and C–O bonds, with a decrease in peak intensity at 1180 cm^−1^ and 1080 cm^−1^, respectively^19^. Moreover, PLLA/ana-TCS nanocomposite showed a little broad peak at 3396 cm^−1^ due to the presence of few free –OH groups. A shoulder peak was observed at 734 cm^−1^ due to vibration of anatase phase Ti–O–Ti bond grafted with end group of PLLA chains^20,21^. An intensified peak assigned to the crystalline phase of PLA chains was found at 758 cm^−1^ due to its incorporation in nanohybrid. All these bonds confirmed the evolution of the structure in the prepared nanohybrid and nanocomposite.

**Figure 2:**
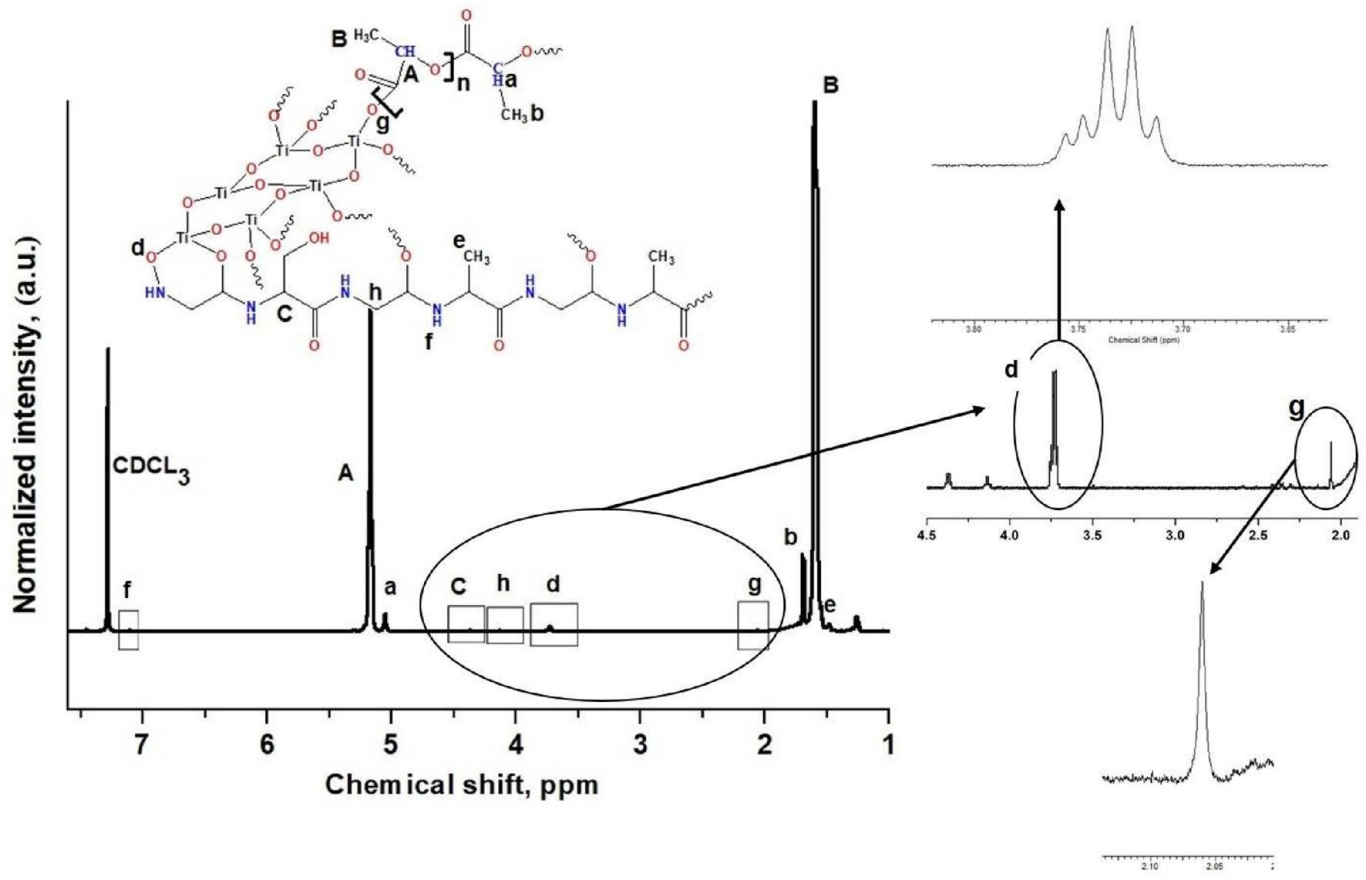
^1^H NMR spectrum of PLLA-g-ana TCS, showing a magnified area within the range of 4.5–1.9 ppm.

The chemical shifts observed in the ^1^H proton NMR spectra have a strong dependence on the intermolecular interactions of chains. CDCl_3_ showed a resonance peak at 7.3 ppm and a little hump peak at 7.1 ppm was seen due to the –NH group of SNC nanoparticles. The –OH end groups of as-TCS played a major role in ring-opening of lactide monomers for their modification. The fabricated PLLA nanocomposite harbored –OH and –COOH functional groups at the two ends of PLLA chains. The resonance signal for the proton of CH_3_ group and CH groups at *δ* = 1.6 and *δ* = 5.2 were assigned to the repetitive units in PLLA. The end group of PLLA chains showed a resonance signal at *δ* = 5, corresponding to the presence of methine group nearby the end –OH group^22,23^. The ^1^H chemical shifts of CH– of Gly, –CH of Ala, and –CH_3_ of Ala were seen at 4.1 ppm, 4.48 ppm and 1.47 ppm, respectively^24^. Thus, the NMR characterization demonstrated fine structure of PLLA chains obtained by grafting its end group chains of –COOH with the TiOH– end groups, giving a resonance peak at 2 ppm. Moreover, another resonance peak at 3.73 ppm with five dips suggested the presence of four hydrogen atoms nearby, signifying the formation of a bond between Ti–OH and C=O of the Gly part of SNC. Figure 2 shows the ^1^H spectra of the prepared PLLA/ana- TCS. A schematic mechanism of grafting has been shown in Supplementary Figure S3, which is similar to the mechanism discussed regarding the grafting of poly(D-lactic acid) and n-HAP^25^.

**Figure 3:**
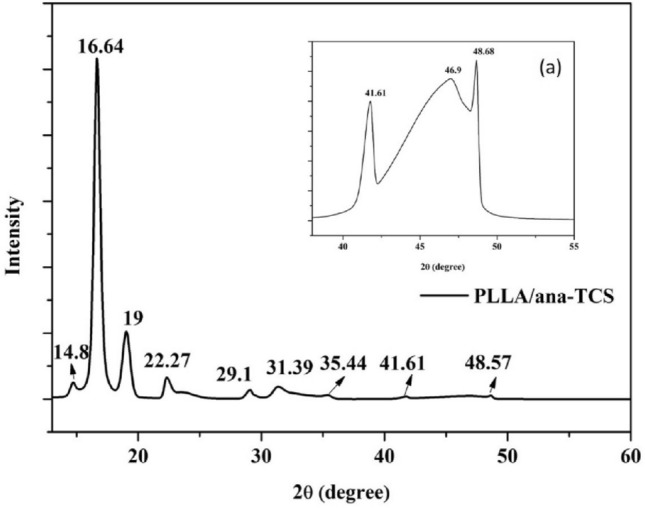
Structural evolution analysis using the XRD spectra of PLLA/ana-TCS, (**a**) inset: the magnified area between the range of 40–50° to show the peak at 48° clearly.

XRD plots were used to confirm the crystallographic structure of the core nanomaterial (SNC) and the nanohybrid obtained after calcination, where area of crystalline peaks/ total area of peaks gives crystallinity percentage (%. SNC showed diffraction peaks at 16.9° (002), 20.3° (201), 24.1° (003), 31° (300), 34.05° (004), 40.0°, and 43.45°, which were attributed to the conformational change from the structure of silk I to silk II, revealing the crystallographic structure of SNC^8,17^. Considering the crystalline peak formula, the crystallinity % of SNC was calculated to be ⁓90% using the Origin Pro 8.5 software. Supplementary Figure S4(a) showed the plot of the as-prepared uncalcined TCS with less intense characteristic peaks observed at 16.8°, 20.4°, 25.4°, 30°, 37.6°, 48°, 54°, and 62.8°, displaying that the amorphous nature of anatase TiO_2_ had covered up the crystalline nature of SNC by engulfing SNC nanoparticles. These peaks became intense after calcination at 300 °C for 3 h. Ana-TCS showed a wide height at 2θ = 25.4°, confirming the formation of the TiO_2_ anatase structure. The other noticeable shifts in the diffraction peaks were observed at 38.08°, 48.0°, 54.35°, 62.8°, 69.7°, and 75.2°, corresponding to different planes, i.e., (101), (104), (200), (105), (204), (116), and (215), respectively, for the calcined anatase phase of TiO_2_ supported over SNC (Supplementary Figure S4(c)). The ana-TCS had a crystallinity percentage of ~ 94%. The d-spacing calculation using Origin Pro 8.5 software have been given in Supplementary Table S1. ^26^. The PLLA chains showed diffraction peaks at 14.7°, 16.7°, and 19.15°, which were attributed to (010), (200/110), and (203) crystal planes (Supplementary Figure S4(b))^27^. The PLLA/ana-TCS had an increased crystallinity percentage of ⁓64% higher than the neat PLLA 30% as reported^28^, due to the formation of a grafted chain with PLLA end groups, confirmed by the overlapping peaks seen in Fig. 3. Eventually, an intense diffraction peak at 16.64° reflected the highly crystalline nature of PLLA nanocomposite, with other peaks being observed at 14.72°, 19.0°, 22.27°, 29.1°, 31.39°, 35.44°, 41.61°, and 48.57°. The less intense, broad diffraction peaks at 22.27°, 31.39°, 35.44°, 41.61° and 48.57° indicated very small-sized crystallite formations. The magnified image within the range of 35° to 55° as shown in Fig. 3a confirmed bond formation by broadening of the peaks between peptide chains and Ti–OH groups.

The average crystal size estimation was done by calculating the full width at half maximum (FWHM) values using the Origin Pro 8.5 software, by selecting the peaks in the XRD plot and applying Debye-Scherer’s formulae “Eq. (1)”. Whereas, the Bragg’s law “Eq. (2)” helped to interpret the interplanar d-spacing between the planes discussed accordingly (Supplementary Table S1)^29^.

1 $$D=\frac{0.9\lambda }{\beta Cos\theta }$$

2 $${\text{2dSin}}\theta \, = \,{\text{n}}\lambda$$

where λ = X-ray wavelength (0.154 nm), $\beta$ = FWHM, ϴ = Bragg’s diffraction angle, D = Particle average diameter, and d = d-spacing.

The bulk anatase TiO_2_ had the following lattice parameters: a = 3.784 Å, c = 9.514 Å, α = β = γ = 90°. The anatase form had a d-spacing of 0.352 nm for the (101) plane, which got shifted to a little higher d-spacing of 0.39 nm, with the 2ϴ value shifting from 25° to 22.27°, confirming chain grafting in PLLA (Supplementary Table S2)^30^.

**Figure 4:**
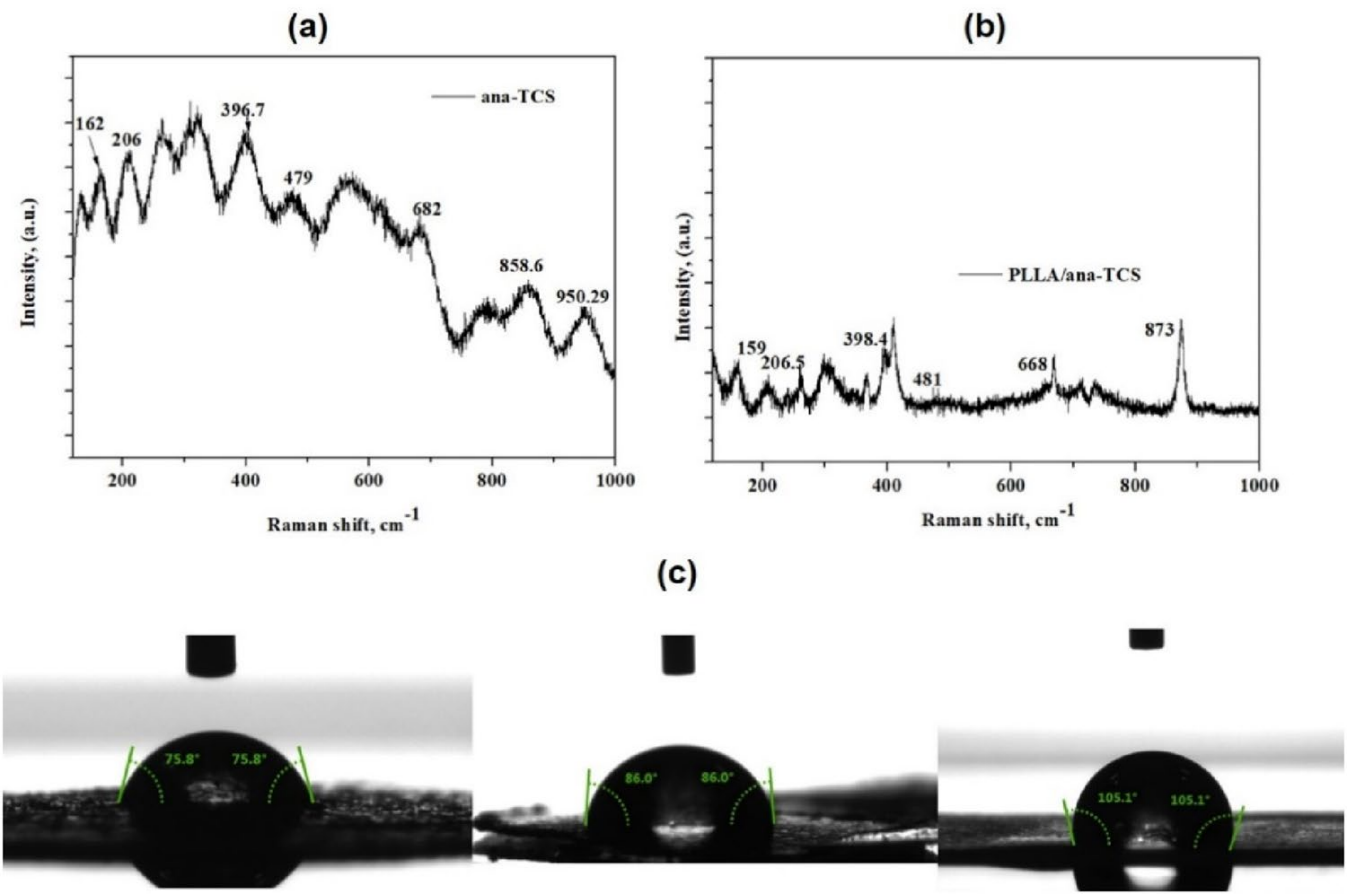
(**a**) Raman spectra of ana-TCS, (**b**) Raman spectra of PLLA/ana-TCS, (**c**) WCA meaasurement of ana-TCS, SNC and PLLA/ana-TCS (from left).

Raman spectroscopic technique was also used for determining the phase of prepared TCS. Figure 4a shows the characteristic shifted Raman peaks observed for ana-TCS nanohybrid. The peaks for SNC were reported to be present at 905 cm^−1^ and 965 cm^−1^, corresponding to the –CN stretching and –CH_3_ rocking, respectively^8^, whereas in case of ana-TCS, the peaks showed a blue shift to 858.6 cm^−1^ and 950.29 cm^−1^, respectively, suggesting formation of the nanohybrid. Basically, anatase TiO_2_ was found to be Raman active in the range of 100–900 cm^−1^. The bands obtained at 162 cm^−1^, 206 cm^−1^, 396.7 cm^−1^, 479 cm^−1^, and 683 cm^−1^ belonged to the well-crystallized anatase form. A red shift of the peaks was observed due to a decrease in the particle size and bond formation between titania and SNC^31,32^. The peaks at 162 cm^−1^ and 206 cm^−1^ were consistent with the Ti–Ti bonding^33^. Moreover, in the case of PLLA/ana-TCS nanocomposite, a sharp peak was seen at 873.6 cm^−1^, which was assigned to the –C=O stretching peak of the PLLA. The Raman plot of the in situ prepared PLLA/ana-TCS showed peak shifting to 160 cm^−1^, 206.5 cm^−1^, 398.4 cm^−1^, 481 cm^−1^, and 668 cm^−1^ due to bond formation between Ti–O–Ti and the C=O bond of PLLA chain end groups as shown in Fig. 4b. ^34^.

### Water contact angle (WCA) analysis

The WCA measurement was performed without any irradiation on a solid surface. The WCA of the nanohybrid taken at 10 s was found to decrease to some extent when TiO_2_ was supported over SNC, i.e., a reduction from 86° to 75.8° was seen due to the presence of –OH end groups on the surface of a complex network formed over titania nanoparticles. But when it was incorporated into the lactide during in situ polymerization, the WCA improved to 105.3° as shown in Fig. 4c, signifying its good hydrophobic nature. PLA has a contact angle of ~ 78° generally, and an increase in the contact angle value for the PLLA nanocomposite was due to the formation of chain linkages between the end groups of PLLA and the surface hydroxyl groups of the ana-TCS as shown by NMR (decrease in open ended chains).

### Morphological analysis

**Figure 5:**
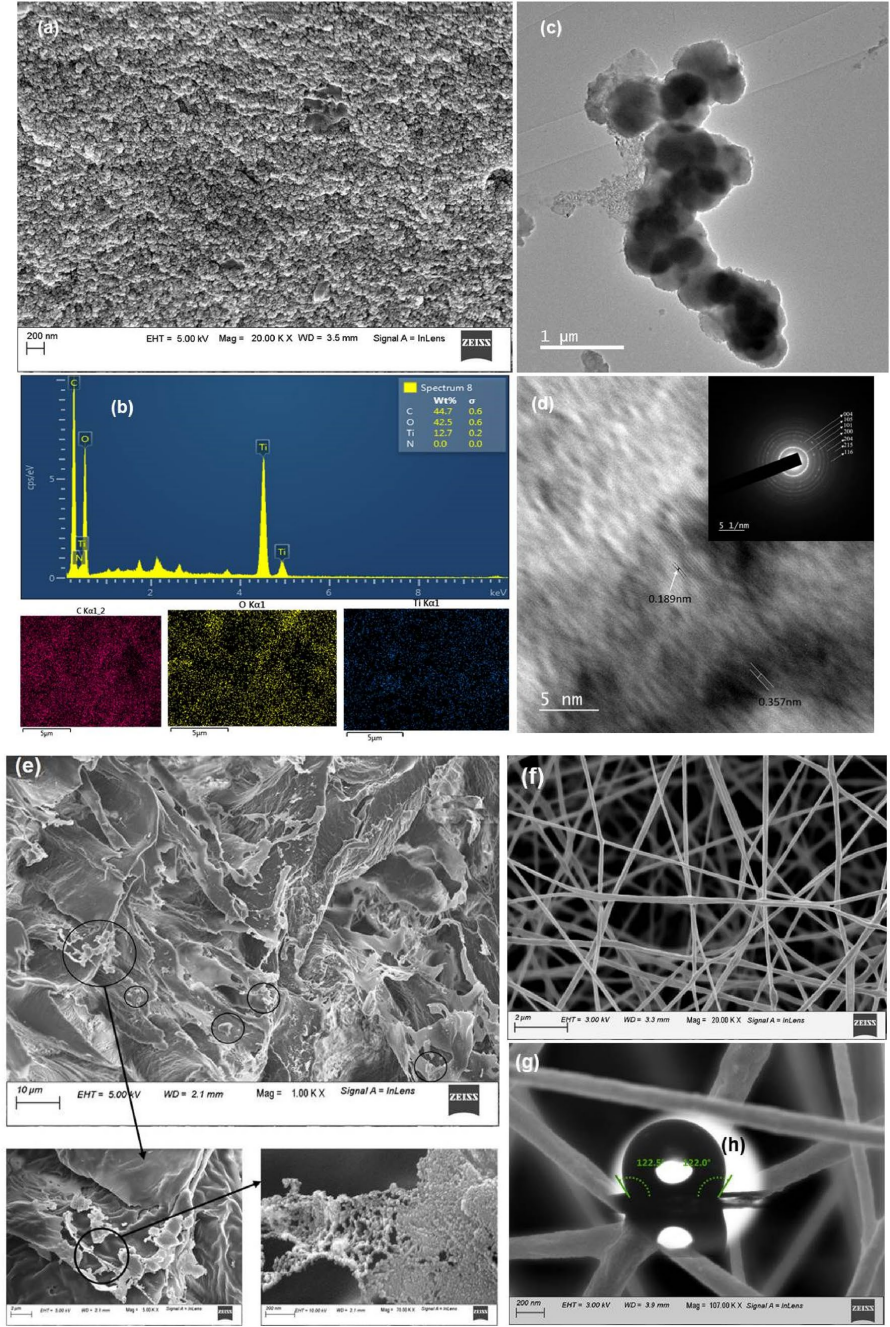
(**a**) FESEM micrograph showing SNC nanoparticles, (**b**) EDX analysis of ana-TCS (inset: mapping for Ti, O, N content with wt. % composition), (**c**) FETEM micrograph of the fabricated ana-TCS, (**d**) HRTEM image elaborating the d- spacing corresponding to the anatase form of TiO_2_ (inset: SAED pattern showing anatase TiO_2_ over SNC template), (**e**) FESEM micrographs of the PLLA/ana-TCS nanocomposite showing the distribution of ana-TCS with network formation of TiO_2_ over SNC nanoparticles in a magnified way, (**f**) PLA/ana-TCS electrospun fiber, (**g**) magnified smooth surface of the electrospun fabric, (**h**) WCA of the nanofabric material depicting surface morphology.

Figure 5a–d shows the morphology of the fabricated nanoparticles, nanohybrid, and their elemental distribution. Figure 5a represents FESEM images of the structural shape of ana-TCS, which revealed a decrease in size of the anatase titania over the surface of SNC as compared to neat SNC on the same scale (Supplementary Figure S5). Figure 5e shows the PLLA/ana-TCS, depicting bond formation between the carboxylic groups in PLLA with TiO_2_ in a bridging bidentate, wherein the spherical titania nanoparticle supported SNC was incorporated in the PLLA matrix.

The composition of the calcined nanohybrid was observed by EDX analysis as depicted in Fig. 5b, which demonstrated the formation of a well grown nanohybrid composed of titanium and oxygen layers. This analysis also showed the presence of 12.7 wt.% Ti. Additionally, elemental mapping analysis helps to investigate the distribution of the specific elements. The absence of SNC wt.% indicated the creation of nanohybrid wherein the SNC nanoparticles were engulfed by Ti–OH bond from all sides. The anatase TiO_2_ nanoparticles with a diameter of 7.5 ± 1.4 nm was found to be doped all over the highly crystalline disc shaped SNC substrate (32 ± 9 nm diameter and 4.2 ± 1.2 nm width) in a well-ordered manner as unveiled by the FETEM images (Fig. 5c). A network formation was observed due to the polycondensation reaction of TiO_2_ nanomaterials over SNC by the sol–gel process. In Fig. 5d, the HRTEM image shows two orthogonal lattice spacings of ~ 0.19 nm, corresponding to the characteristic d_200_ and d_020_ planes of anatase titania, and 0.35 nm for the d_101_ plane^35^. On the other hand, SAED patterns revealed the visibility of highly polycrystalline circular rings for (101), (004), (200), (105), (204), (116) and (215) planes, corresponding to the anatase phase of titania. The calculated d-spacing value was matched with the value for anatase TiO_2_ e phase having the same lattice planes. Incorporation of nanohybrid during the in situ polymerization of PLLA resulted in its network kind of distribution in the polymer matrix, as seen clearly in Fig. 5e. From Fig. 5f,g, it was observed that the electrospun fibers formed a rough surface, having an average diameter of 178 ± 30 nm. This surface property further enhanced the hydrophobicity of the prepared nanofabric with a WCA of 122°. The average dimensional calculations were done using Image J® software.

### Thermal analysis

The thermal behavior of the degummed SF, SNC, TCS, ana-TCS, PLLA/ana-TCS are shown in (Supplementary Figure S6a). In the case of nanomaterials and nanohybrid, peaks observed below 120 °C were due to the elimination of adsorbed water. The char residue enhanced from 3.9 to 54% at 700 °C in case of ana-TCS, which might be ascribed to the presence of inorganic anatase TiO_2_ doped over SNC, suggesting no further dissociation of carbonaceous material with an enhancement in thermal stability. The TGA thermogram of the nanocomposite showed maximum degradation at 320 °C for the PLLA chains and left back a char residue of 0.37% due to the incorporation of 0.5 wt. % of ana-TCS. This little change in the char value even upon incorporation was because of the grafting of the chains with PLLA end groups wherein the grafting % was found to be 26%.The TGA plot also suggested the successful synthesis of PLLA nanocomposite via in situ polymerization technique.

The PLLA chains crystallized in α form when T_c_ > 120 °C, wherein the melting and crystallization behavior was mostly related to chain entanglement. Figure S6b shows the 2nd heating plot of DSC, which gave the values of T_g_ as 60 °C, T_cc_ as 125 °C, ΔH_cc_ as 165.2 J/g, endothermic peak with melting temperature as 173 °C and ΔH_m_ as − 199.2 J/g.

### Sperulite growth analysis

**Figure 6:**
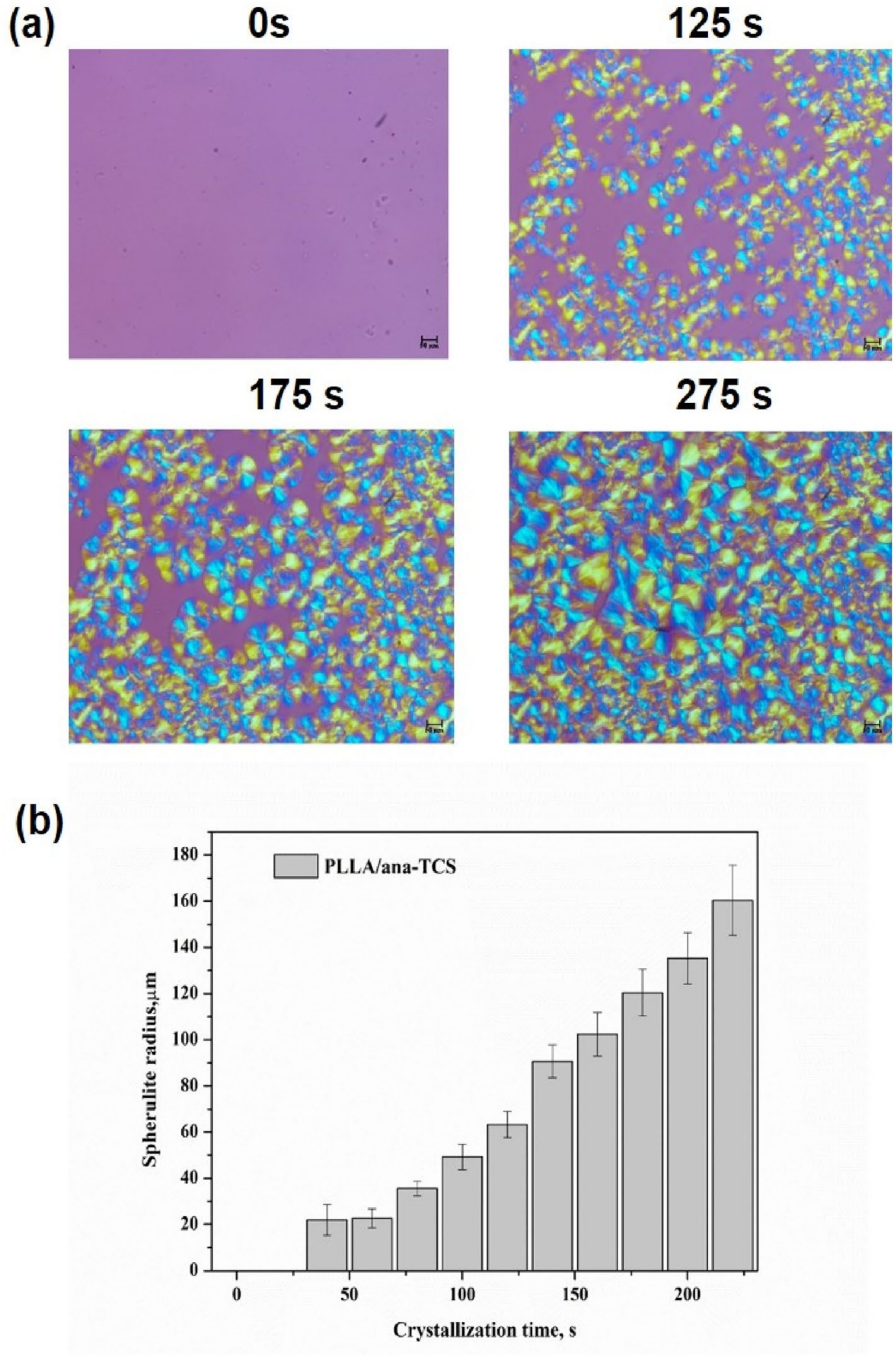
(**a**) POM images showing spherulite growth formation in the nanocomposite at isothermal condition of 120 °C, (**b**) Histogram plot showing spherulite growth rate in the nanocomposite with increasing crystallization time.

PLLA showed a characteristic slow crystallization behavior, and therefore the presence of nucleating agents might induce the growth of crystals. The presence of long grafted compatible nanofillers throughout the polymer matrix regulated the rate of nucleation and the density of spherulites, by accelerating the crystallization process. POM images of the isothermally crystallized PLLA/ana-TCS nanocomposite showed a maltese cross pattern (Fig. 6a). As time escalated, there was an increase in the size of the spherulites very quickly, covering the whole area in only 275 s. It might be due to the presence of grafted chains which induced crystallinity, filling up the free volume, thereby overlapping the nearby spherurites, suggesting high crystallinity of the nanocomposite. Figure 6b shows the POM micrographs of the PLA-based ana-TCS nanocomposite at different time intervals. The average diameter of the full-grown spherulites were found to be 160 $\pm$ 15 µm in 220 s.

### Photocatalytic activity analysis

**Figure 7:**
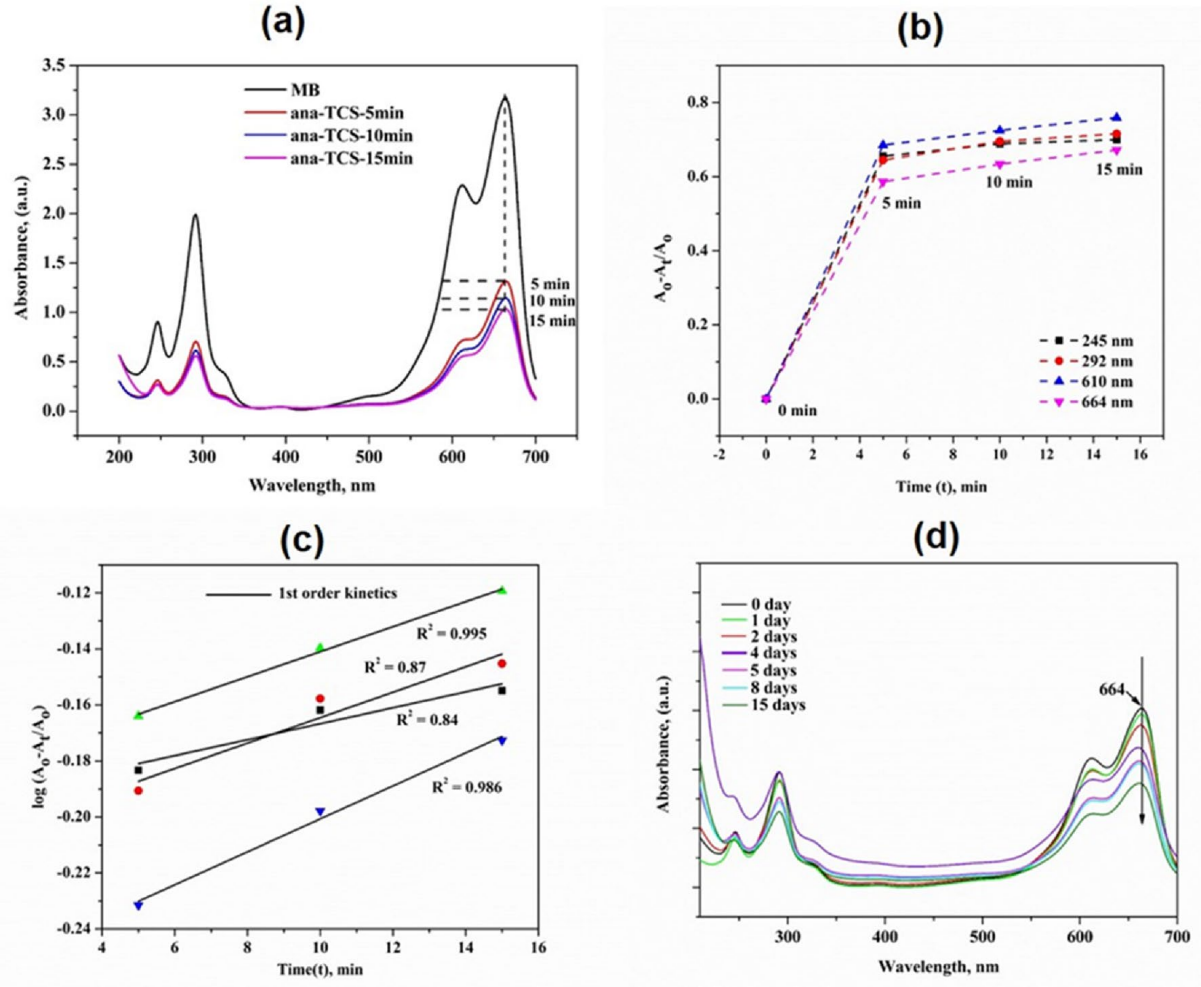
(**a**) Photocatalytic activity of ana-TCS in organic dye (MB), (**b**) Discoloration plot showing absorbance change with time, (**c**) Pseudo-first order kinetics for the 664 nm peak wherein R^2^ ⁓ 1, (**d**) MB discoloration plot for the PLLA/ana-TCS nanocomposite with time for the peaks 610 nm and 664 nm.

To evaluate the experimental parameters obtained due to catalytic photodegradation, UV–vis spectroscopy was used to determine the decrease in band intensity at wavelength peaks of 245, 292, and 610 nm, and at a maximum peak of 664 nm with an increase in time. Figure 7a–d show that when anatase TiO_2_ nanoparticles were doped over SNC nanoparticles, the dye started to decompose MB at a quick rate. This could happen due to the vigorous oxidizing activity of the photo-generated holes or due to OH^.^ groups^36^. The reason behind this was the position of the conduction band such that it either reduced the oxygen molecules present or got adsorbed on the surface of MB. Therefore, due to the adsorption of MB over metal oxide, the blue coloration of the solution faded away after 15 days of time under UV illumination. The chromophores present in MB were mainly responsible for the discoloration of the solution^37^. The mechanism that took place due to the degradation of MB dye resulted in the generation of conduction band (e^−^) electrons and valence band (h^+^) holes when ana-TCS was irradiated with light energy greater than or equal to its band gap energy. These holes and electrons mediated the oxidation of organic compounds to (OH•) radicals and (O2•-) superoxide radicals. The (OH^•^) oxides then degraded most of the MB colorant into non-toxic products^38^.

A parallel study was done for the in situ synthesis of PLLA using nanohybrid so that the PLLA nanocomposite could act as a catalyst for dye removal when exposed to UV irradiation (Supplementary Figure S7). Figure 7d shows a decrease in the degradation of MB with time, observed because of a noticeable reduction in peak position at 664 nm.

For calculating the percentage degradation under UV irradiation, the ana-TCS values were taken and absorbance was calculated for all the humps, following the modified equation “Eq. (3)” of Beer-Lambert Law.

3 $$\mathrm{\%Degradation}=(1-\frac{{A}_{t}}{{A}_{o}})\times 100$$

where A_0_ = initial absorbance of dye at time 0 min, and A_t_ = absorbance of dye after time t min.

Similarly, the kinetics of the photodegradation rates of most of the organic contaminants followed pseudo-first-order kinetics as given by the following equation “Eq. (4)”.

4 $$- {\text{dA}}/{\text{dt }} = {\text{k}}_{{{\text{obs}}}} {\text{A}}$$

where A = absorbance of MB, k_obs_ = observed value for pseudo first-order kinetics after integrating it from A_o_ to A_t_. The R^2^ value was found to be almost equal to 1 for the peaks observed at 610 and 664 nm other. (Fig. 7 (c)) depicts the best fitted equation. Thereby, the peak at 664 nm was considered for calcultions as it had R^2^ = 0.995, using which the dye degradation of MB was found to be 43%.

### Effect of MB adsorption under neutral pH

To evaluate the adsorption of MB cations on the surface of the prepared nanocomposite, the change in weight of the material, and molecular weight change after the 15th day was noted, by dipping the piece into MB solution under UV light (pH = 7). For reference, the degradation of neat PLLA was also checked under neutral pH.

Neat PLLA degradation was studied by dipping the polymer in DI water for 15 days and noting down the readings. It could be seen from (Supplementary Figure S8a–d) that there was very less change in the weight of neat PLLA at pH 7. The change in molecular weight for neat PLLA was calculated and it was found that almost 25% polymer had been degraded. But, when PLLA/ana-TCS nanocomposite was treated with MB for its discoloration under UV irradiation, 3% of adsorbate was adsorbed on the surface of PLLA/ana-TCS due to the presence of Ti–OH group which reinforced its self-cleaning property (Figure S8c). Moreover, the molecular weight value of the nanocomposite was found to be decreased by 50%, suggesting increased degradation rate for the prepared PLLA nanocomposite in comparison to the neat PLLA, which would further lead to the development of a sustainable environment.

## Conclusion

In the present study, a single-phase SNC templated anatase TiO_2_ (ana-TCS) nanohybrid was successfully prepared by a novel sol–gel process in an aqueous media, followed by aging at room temperature to promote crystallization. A crystalline anatase phase network-like structure of TiO_2_ engulfing SNC was found to form after post-treatment at high temperature, resulting in the formation of ana-TCS nanohybrid. Furthermore, incorporation of this nanohybrid as a co-initiator during ring-opening polymerization of lactide rings via an in situ polymerization process, subsequently led to the fabrication of high molecular weight PLLA grafted ana-TCS nanocomposite. As a result of the inclusion of ana-TCS into the PLLA matrix, the crystallinity as well as the thermal stability of the nanocomposite increased. The prepared PLLA-g-ana-TCS nanocomposite presented a highly hydrophobic surface to remove dye and acted as a self-cleaning material. XRD and POM analysis showed that the prepared nanocomposite was a crystalline material, showing fast spherulite growth. The work also showed that the prepared nanohybrid could act as a nucleating agent under isothermal conditions. The fabricated ana-TCS/PLLA nanocomposite also promoted significant photocatalytic tendency within 15 days of time by degrading 43% of the organic dye, MB under UV irradiation at neutral pH. Additionally, the PLLA component in the nanocomposite underwent no significant hydrolytic degradation when dipped in an aqeous solution for 1 h. Thus, the developed nanofabric could be promoted as a degradable, self-cleaning cloth material which could be a way forward to tackle the serious environmental problems of discard of textiles in both landfills as well as in marine regions by industries, and which could also be used for waste water treatment.

## Materials and methods

### Materials

L-lactic acid was purchased from Purac, India. Synthesized PLLA as reported elsewhere^27^ have high optical purity ([Optical purity = − 157]^T=25°C^_λ=589 nm_). The waste cocoons (*Antheraea assama),* popularly known as Muga silk, received from Regional Muga Silk Station (Central Silk Board, Assam, India), was used as SF source, prepared by the acid hydrolysis technique using sulfuric acid (H_2_SO_4_)^8^. The precursor, titanium (IV) butoxide (TTB) (99% purity) and absolute ethanol (used without further purification as a solvent), were purchased from Aldrich Chemical Co., USA. Distilled water (DI), Nitric acid (HNO_3_) (69% purity) (HiMedia Laboratories Pvt. Ltd., India), Analytical grade chemicals like sodium carbonate (Na_2_CO_3_) (> 99% purity), H_2_SO_4_ (> 99% purity), and methylene blue (MB) were procured from SRL Chemicals, India. Chloroform (HPLC grade), was acquired from Spectrochem, India and N,N-dimethyl formamide (Emplura®,Merck, India).

### ***Fabrication of TiO***_***2***_*** doped SNC nanohybrid***

Hydrolysis of the alkoxide precursor TTB was carried out in an alcoholic medium using the acid catalyst HNO_3_ as a hydrolysis agent. For sol–gel process, firstly 0.1 g SNC, 2.5 mL HNO_3_ and 50 mL absolute ethanol were kept in a sonication bath for 1 h to promote uniform dispersion of SNC in the medium. Then, placed in a one-neck round bottom flask (RB) for 4 h, maintained at a temperature of 45 °C. 1 mL TTB was added drop wise with continuous stirring until it turns to a white gel-like in appearance^39^. Then fivefold times DI water was added to stop any further reaction and centrifuged using DI water at 6000 rpm for 15 min to obtain a slurry. Thereafter, dialysis was performed as shown in (Supplementary Figure S1). The presence of -NH group in SNC chains might have resulted in change in color. Using cellulose acetate membrane (Sigma-Aldrich, India) it was dialysed in DI water for 3 days to obtain neutral pH. The freeze-dried (− 100 °C) sample was dried at 70 °C for 2 h for aging to promote the crystallization of anatase phase, and calcined at 300 °C (below degradation temperature of SNC) for 3 h to obtain pure crystalline anatase-TiO_2_ powder.

### Fabrication of PLLA/a-TCS nanocomposite by in situ polymerization

The nanocomposite was prepared by in situ polymerization. L-lactide, (moisture content = 0.06 ppm), a two-neck RB was taken. One neck of RB flask was connected to high purity (99.99%) nitrogen gas and the temperature was raised to 50 °C for an hour. Then tin-octoate, monomer/Catalyst [M/C] = 2500/1) was loaded into the RB using a 250 µL syringe, 15 g L-lactide was added under nitrogen atmosphere. Next, 0.5 wt. % of the prepared ana-TCS powder was loaded into the RB that act as co-initiator, where coordination insertion mechanism leads to opening of L-lactide rings. The temperature was raised to 180 °C at a speed of 400 rpm and the reaction was continued for 30 min till a solid mass was obtained. The material was then kept in an oven at 40 °C for 24 h to remove any moisture content for further analysis.

### Processing of PLLA/ana-TCS nanocomposite by electrospinning

Further the electrospinning technique was used to fabricate the prepared nanocomposite into a non-woven nanofibrous mat over aluminum (substrate). 10% (wt./vol.) PLLA/ana-TCS solution was prepared in a chloroform: DMF (70:30) solvent mixture stirred at 50 °C for 6 h. Parameters used during electrospinning were as follows: 12 kV voltage, tip distance from the collector was maintained at 12 cm using a 6 mL syringe for spinning the solution to the collector. The electrospun nanofibers were then dried in a vacuum oven at 40 °C for 12 h.

### Characterization

Gel permeation chromatography (GPC) system equipped with a refractive index detector (Shimadzu, Japan) was used to determine the molecular weight at 1 mL min^−1^ flowrate, at a temperature of 40 °C.

For intermolecular interaction identification, diffused reflection spectroscopic (DRS) method was performed for powder using FTIR (Shimadzu, Japan) from 4000–400 cm^−1^ with 8 scans. For films, attenuated total internal reflection (ATR) mode within range of 650–4000 cm^−1^ with 4 cm^−1^ resolution and 16 scans was used.

20 mg mL^−1^ nanocomposite in deuterated chloroform (CDCl_3_) was taken and filtered with 0.25 μm PTFE syringe filter. The proton ^1^H NMR spectra was recorded in a 600 MHz NMR spectrometer (ASCEND 600), at 500 scans in spectral range of 0–10 ppm to support grafting.

To identify isotactic polyenantiomers of PLA, (100 mg mL^−1^) in chloroform were prepared using AUTOPOL II polarimeter (Rudolph Research Laboratory) at wavelength of 589 nm.

X-ray diffraction (XRD) diffractometer by SmartLab® X-ray diffraction system (TTRAX III 18 kW, Rigaku, Japan), gives crystal structure with Cu − Kα radiation wavelength, λ = 0.1541 nm, at (40 kV, 40 mA) at a scan rate of 5° per min, within a 2θ range of 5°-80°.

For structural fingerprint raman spectrophotometer (LabRam HR; Horiba Jobin Vyon), equipped with a 1-W, Nd: YAG with a diode-pumped laser was used. The excitation wavelength is 785 nm, 1024 scans, 10 s exposure time with 50X magnification.

To perform the water race analysis, water contact angle measurement was done using a goniometer (DSA-25 Expert model, Kruss). Samples were glued to a glass slide with a double-sided transparent tape. The instrument set to 27 °C where 2 μL of DI water was dropped at rate of 0.16 mL min^−1^ using syringe. This specimen was captured using video mode, and the measurement was made in 10 s using Young’s Laplace equation.

The sample’s internal crystalline bulk morphology was studied using a high resolution transmission electron microscope (HRTEM) (JEM-2100) at 200 kV. Powder samples were drop-casted (0.01 wt.%). For the nanocomposite, samples were sliced using a microtome (Leica microsystem), which were then placed onto a carbon coated copper grid of 300 nm mesh size and left overnight in oven for drying. Images were taken at high resolution and at a magnification of 5 nm scale. The selected area electron diffraction (SAED) patterns for the samples were captured for determining the crystalline rings.

The topographical view of the samples was studied using a field emission scanning electron microscope (FESEM) (Sigma-Zeiss) at an accelerated voltage of 2–4 kV. The samples were treated in a conductive gold sputtering unit for 30 s before analysis. 0.01 wt. % suspension was sonicated for 10 min, and then drop-casted. Whereas, 0.01 g of the sliced nanocomposite pellet, fabric was directly placed over a carbon black tape.

To examine the elemental composition of the ana-TCS sample, energy dispersive X-ray (EDX) spectroscopy (Oxford Instruments), operated at ~ 20 kV, was used. The powdered samples (⁓2 mg) were floated over carbon black tape and blown over with an air blower to spread the treated sample and gold sputtering over it was done for 30 s before analysis.

To determine optical images for spherulite growth, POM (Eclipse LV100N POL, Nikon Co. connected to a hot stage set-up (Instec TST350, Linkam Scientific Instrument) wa used. 0.001 g sample was sandwiched between two cover slips over hot stage, maintaining isothermal conditions. The sample was melted up to 190 °C for 3 min and maintained at an isothermal temperature of 120 °C for 7 min by cooling at a rate of 10 °C min^−1^. Images were captured at an interval of every 5 s by a digital camera.

The thermal behaviour were examined using a differential scanning calorimeter (DSC) (Phoenix-DSC 204, F1 NETZSCH) in under nitrogen atmosphere. ⁓ 7.5 mg of sample was placed in the platinum crucible, and the program was set for heating from 20 to 200 °C, and then kept under isothermal conditions at 200 °C for 2 min to wipe out the thermal history. The material was then cooled to 20 °C at a rate of 10 °C·min^−1^. It was then again heated to 200 °C, maintaining the same rate. The curves thus obtained gave the values of glass transition temperature (T_g_), cold crystallization temperature (T_cc_), melting temperature (T_m_), enthalpy of crystallization (H_cc_), and enthalpy of fusion (H_m_), of the samples.

The thermal degradation were analysed using a thermogravimetric analysis (TGA) instrument (TGA4000, PerkinElmer). ⁓ 6.5 mg of sample was taken and heated from 30 to 700 °C at nitrogen flow rate of 20 mL min^−1^.

The dye degradation and photocatalytic activities of the fabricated samples were studied in a UV–Vis spectrophotometer (Perkin–Elmer) in the wavelength range of 200–700 nm using MB by checking the color reduction for 20 mg L^−1^. For the analysis, the fabricated nanocomposite was placed in a 5 mL MB solution in a beaker. The beaker was then placed inside a UV chamber with a 8 W T5/ backlight blue UV tube.

## Supplementary Information

Supplementary Information.

## Data Availability

All data generated or analysed during this study are included in this published article (and its Supplementary Information files).
