# Supplementary material for: Structural evolution of in situ polymerized poly(L-lactic acid) nanocomposite for smart textile application
Source: Sci Rep. 2022 Aug 30;12:14724. doi: 10.1038/s41598-022-17437-z (PMC9427861; doi:10.1038/s41598-022-17437-z)
Supplement: Supplementary file 1 — Supplementary Information. [file 41598_2022_17437_MOESM1_ESM.docx]

**Supplementary Information**

**Structural Evolution of *in situ* Polymerized Poly(L-lactic acid) Nanocomposite for Smart Textile Application**

**Doli Hazarika^1^, Amit Kumar*^1^, Vimal Katiyar*^1^**

*^1^Department of Chemical Engineering, Indian Institute of Technology Guwahati, Assam-781039, India*

**Corresponding author’s e-mail:* [*vkatiyar@iitg.ac.in*](mailto:vkatiyar@iitg.ac.in) *and* [*amitkumar@iitg.ac.in*](amitkumar@iitg.ac.in)

**Table S1:** Calculation of average diameter, D and d-spacing for ana-TCS from XRD spectra.

| **Sl. No.** | **2ϴ**  **(°)** | **FWHM**  **(°)** | **ϴ**  **(°)** | **Cos ϴ**  **(°)** | **FWHM**  **(radian)** | **D**  **(nm)** | **Sin ϴ**  **(°)** | **d-spacing**  **(nm)** |
| --- | --- | --- | --- | --- | --- | --- | --- | --- |
| **1** | **25.40** | **2.41** | **12.70** | **0.97** | **0.017** | **3.37** | **0.22** | **0.35** |
| **2** | **38.20** | **0.11** | **19.10** | **0.94** | **0.016** | **76.03** | **0.33** | **0.23** |
| **3** | **48.10** | **1.15** | **24.10** | **0.91** | **0.016** | **7.55** | **0.41** | **0.19** |
| **4** | **54.50** | **2.24** | **27.20** | **0.89** | **0.015** | **3.98** | **0.45** | **0.17** |
| **5** | **62.80** | **1.59** | **31.40** | **0.85** | **0.014** | **5.84** | **0.52** | **0.15** |
| **6** | **75.20** | **0.31** | **37.60** | **0.79** | **0.015** | **32.50** | **0.61** | **0.14** |

**Table S2:** Calculation of average diameter and d-spacing from the XRD spectra for PLLA/ana- TCS.

| **Sl. No.** | **2ϴ**  **(°)** | **FWHM**  **(°)** | **ϴ**  **(°)** | **Cos ϴ**  **(°)** | **FWHM**  **(radian)** | **D (nm)** | **Sin ϴ (°)** | **d-spacing (nm)** |
| --- | --- | --- | --- | --- | --- | --- | --- | --- |
| 1 | 14.7 | 0.83 | 7.36 | 0.990 | 0.014 | 9.7 | 0.13 | 0.60 |
| 2 | 16.6 | 0.60 | 8.32 | 0.989 | 0.010 | 13.5 | 0.14 | 0.53 |
| 3 | 19.0 | 0.73 | 9.52 | 0.986 | 0.013 | 11.1 | 0.16 | 0.46 |
| 4 | 22.2 | 0.70 | 11.16 | 0.981 | 0.012 | 11.5 | 0.19 | 0.39 |
| 5 | 29.0 | 0.63 | 14.54 | 0.968 | 0.011 | 12.9 | 0.25 | 0.31 |
| 6 | 31.4 | 1.57 | 15.70 | 0.963 | 0.027 | 5.2 | 0.26 | 0.29 |
| 7 | 35.4 | 1.04 | 17.70 | 0.953 | 0.018 | 8.0 | 0.30 | 0.25 |
| 8 | 41.8 | 0.74 | 20.90 | 0.934 | 0.013 | 11.5 | 0.35 | 0.21 |
| 9 | 48.7 | 4.80 | 24.34 | 0.911 | 0.084 | 1.81 | 0.41 | 0.19 |

**
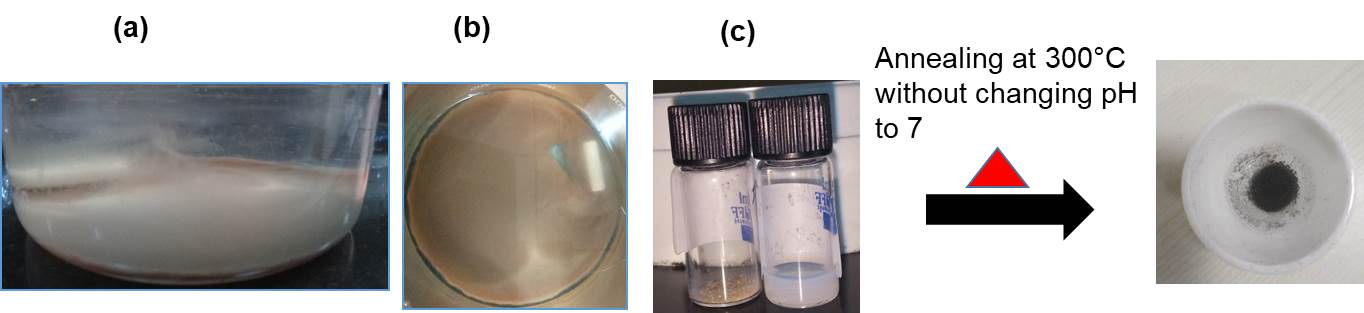
**

**(d)**

**Figure S1:** (a) Ana-TCS in DI water, (b) Aged Ana-TCS (c - left) dried powder, (c - right) in DI water after drying to check for any visual changes after aging, (d) Change in color seen after annealing without neutralizing the nanohybrid.

**

**

**Figure S2:** GPC chromatogram of the PLLA/ana-TCS nanocomposite.

**
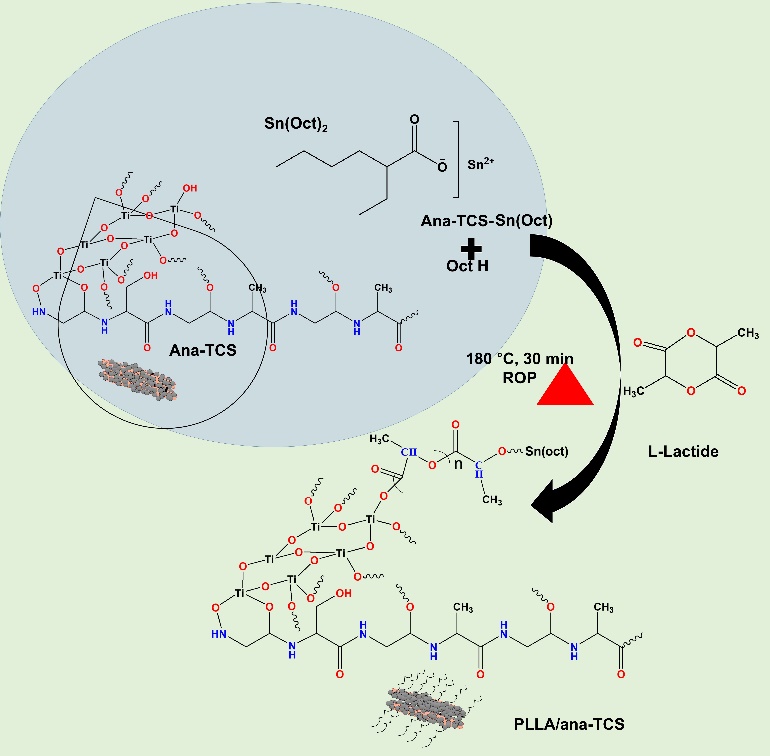
**

**Figure S3:** Schematic diagram depicting grafting of ana-TCS with PLLA

**
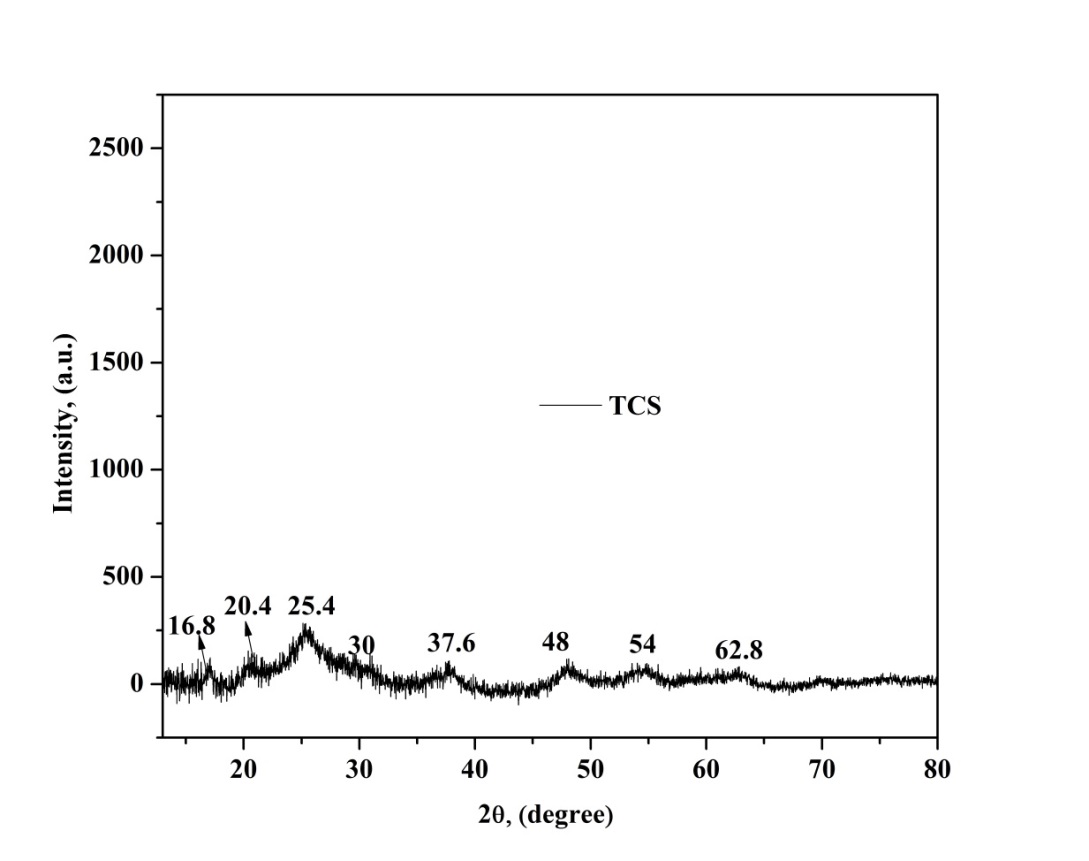
**

**(a)**

**
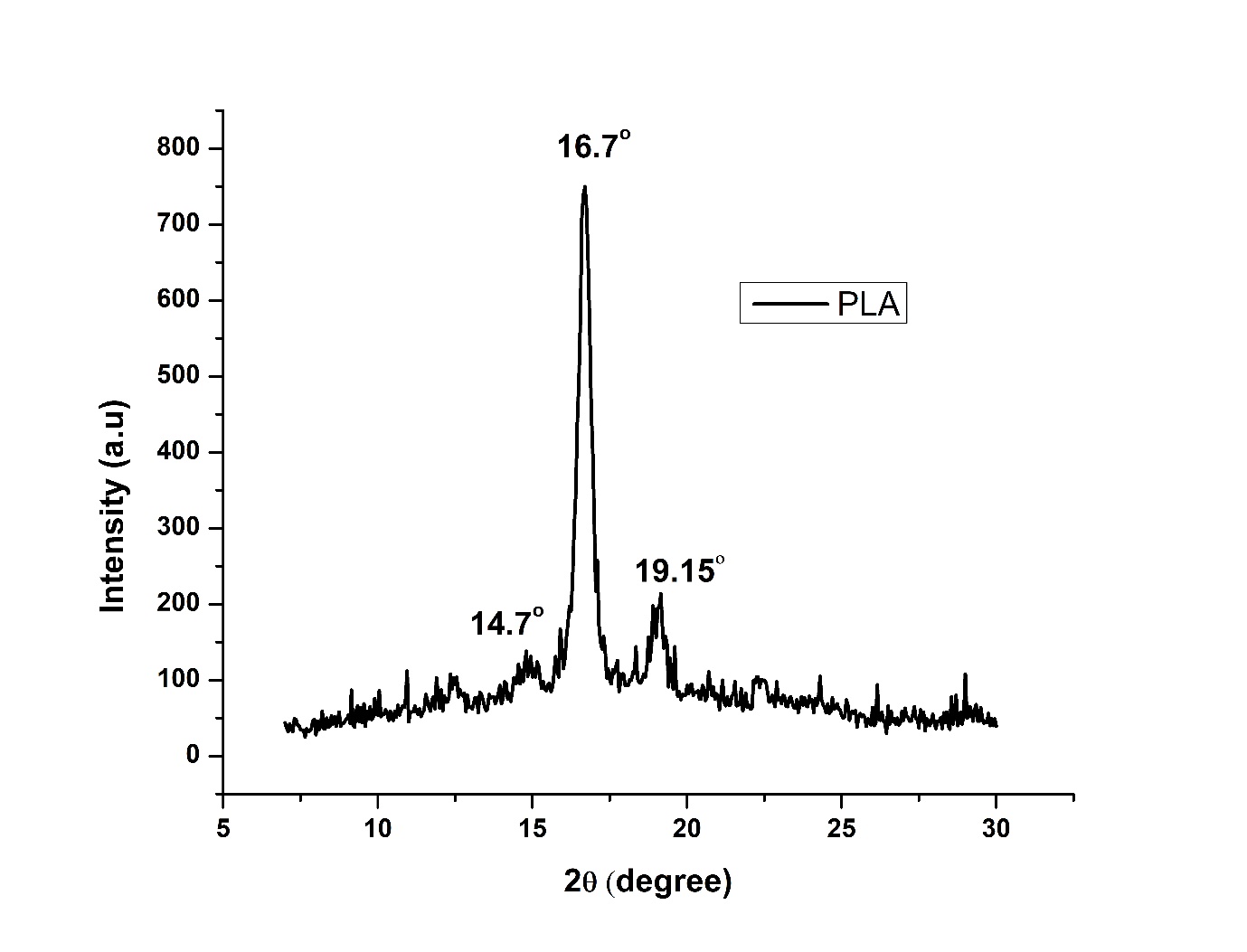
**

**(b)**

**
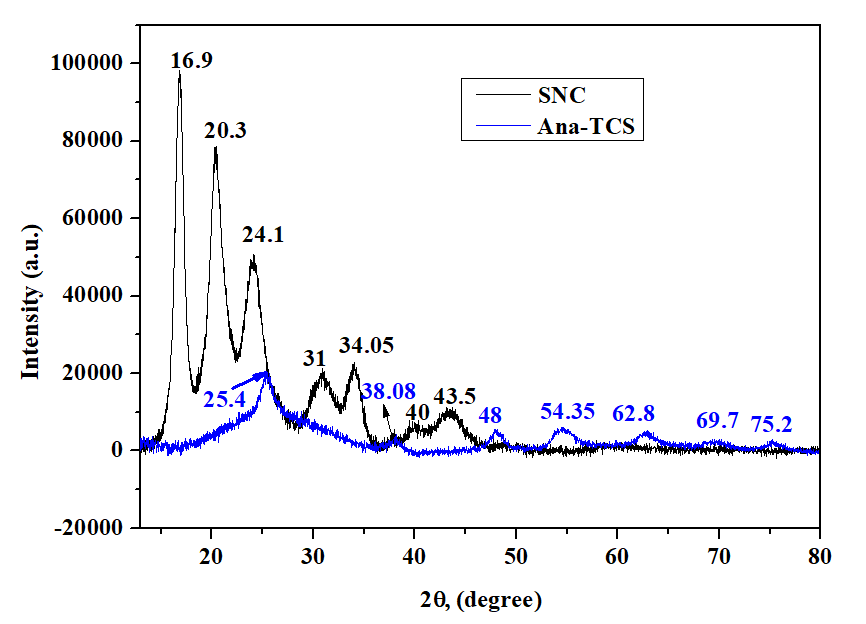
**

**(c)**

**Figure S4:** XRD spectra of the (a) prepared TCS, (b) PLLA, (c) SNC and ana-TCS (calcined at 300 °C).


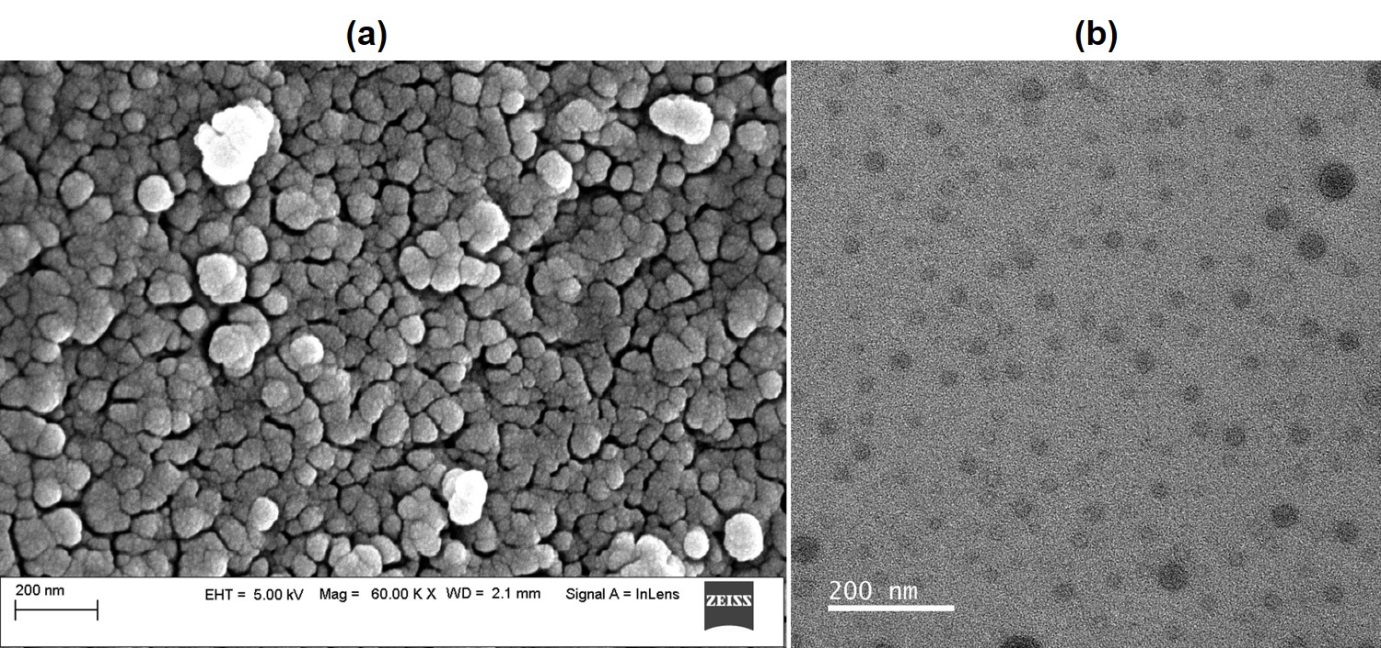


**Figure S5:** (a) FESEM and (b) FETEM images of SNC distribution at 200 nm scale.


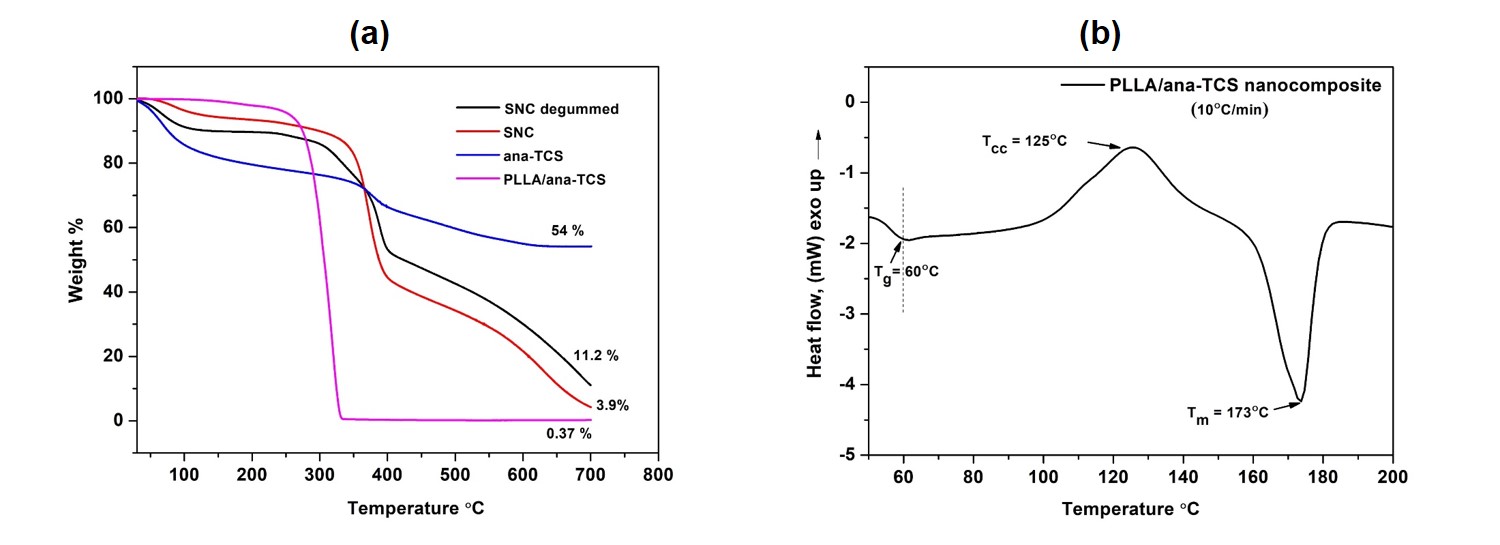


**Figure 6:** (a) TGA analysis plot of the SNC, ana-TCS and PLLA/ana-TCS nanocomposite, (b) DSC thermograms showing T_g_, T_cc_ and T_m_ of the PLLA/ana-TCS nanocomposite.


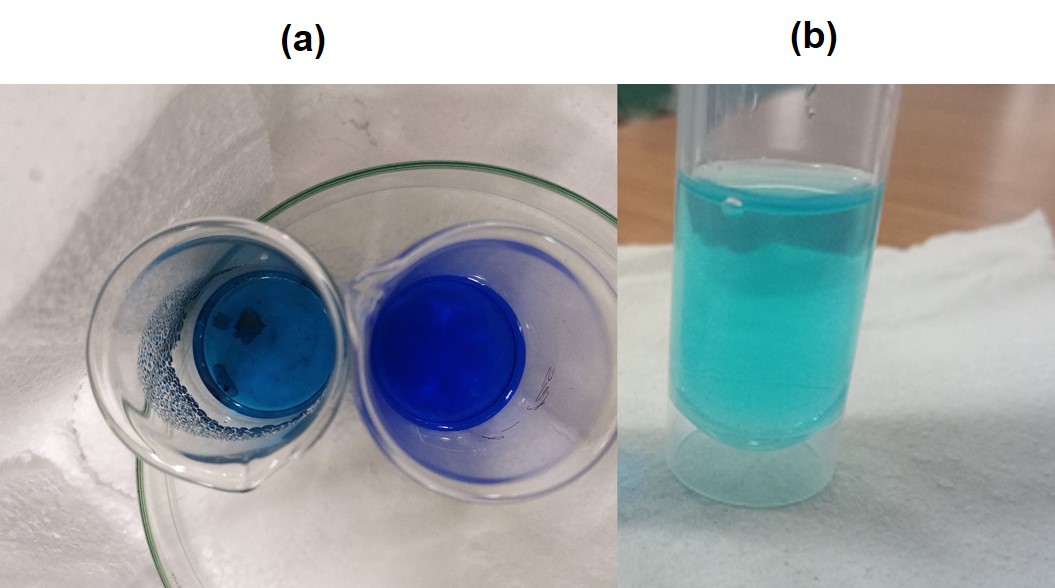


**Figure S7:** Digital images showing photocatalytic degradation of MB by (a) PLLA/ana-TCS as compared to crude MB and (b) ana-TCS nanohybrid.


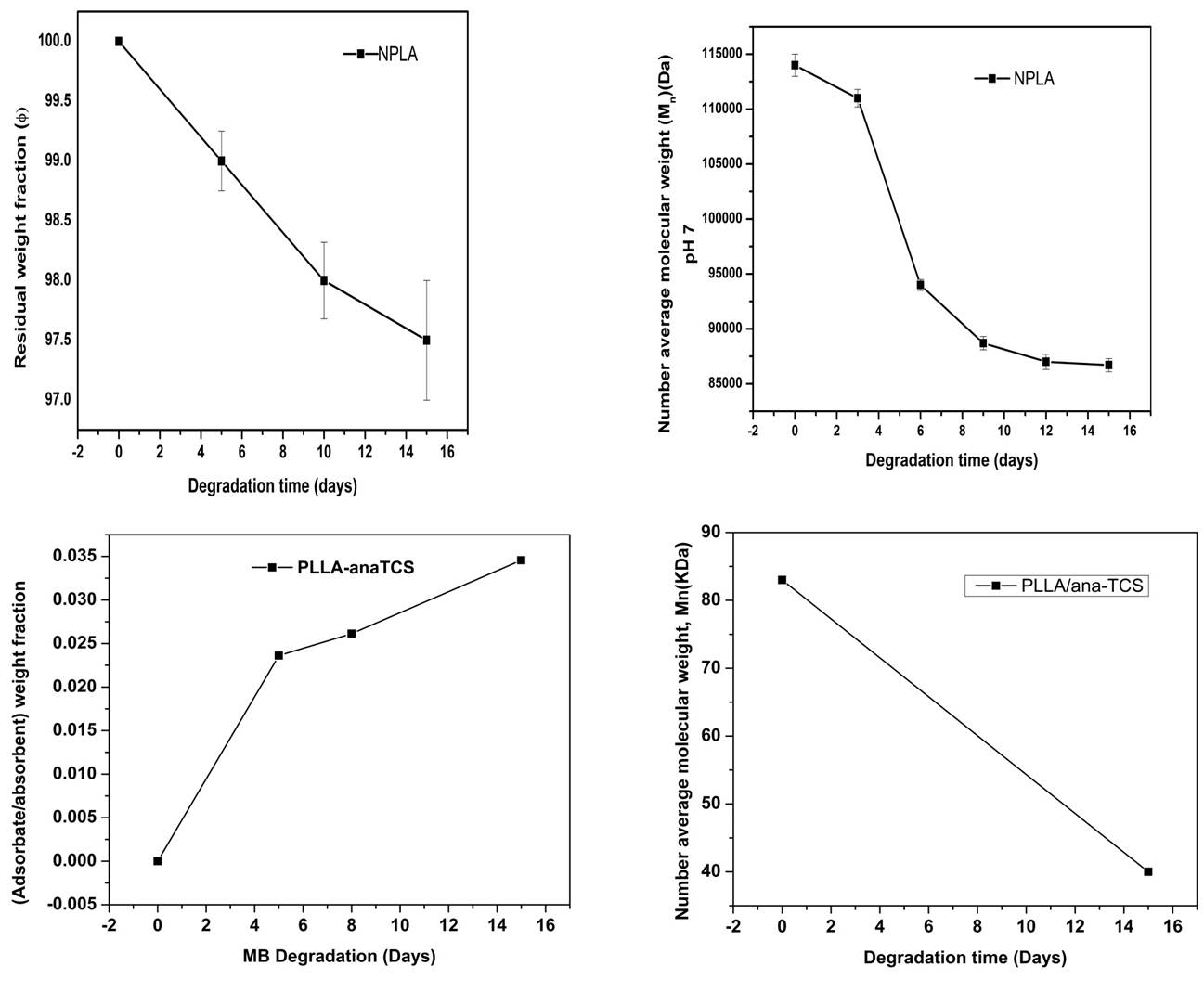


**(b)**

**(a)**

**(d)**

**(c)**

**Figure S8:** (a) Change in residual weight fraction of PLLA kept in water for 15 days for degradation, (b) Number average molecular weight vs. Degradation time of PLA, (c) Adsorbate/Adsorbent vs. MB degradation plot, (d) Number average molecular weight vs. Degradation time of PLLA/ana-TCS nanocomposite.
